# A Mouse Model for Osseous Heteroplasia

**DOI:** 10.1371/journal.pone.0051835

**Published:** 2012-12-19

**Authors:** Michael T. Cheeseman, Kate Vowell, Tertius A. Hough, Lynn Jones, Paras Pathak, Hayley E. Tyrer, Michelle Kelly, Roger Cox, Madhuri V. Warren, Jo Peters

**Affiliations:** 1 Medical Research Council Mammalian Genetics Unit, Medical Research Council Harwell, Oxfordshire, United Kingdom; 2 Mary Lyon Centre, Medical Research Council Harwell, Oxfordshire, United Kingdom; 3 Pathology Diagnostics Ltd, Cambridge, United Kingdom; University of Leuven, Belgium

## Abstract

*GNAS/Gnas* encodes G_s_α that is mainly biallelically expressed but shows imprinted expression in some tissues. In Albright Hereditary Osteodystrophy (AHO) heterozygous loss of function mutations of *GNAS* can result in ectopic ossification that tends to be superficial and attributable to haploinsufficiency of biallelically expressed G_s_α. *Oed-Sml* is a point missense mutation in exon 6 of the orthologous mouse locus *Gnas*. We report here both the late onset ossification and occurrence of benign cutaneous fibroepithelial polyps in *Oed-Sml*. These phenotypes are seen on both maternal and paternal inheritance of the mutant allele and are therefore due to an effect on biallelically expressed G_s_α. The ossification is confined to subcutaneous tissues and so resembles the ossification observed with AHO. Our mouse model is the first with both subcutaneous ossification and fibroepithelial polyps related to G_s_α deficiency. It is also the first mouse model described with a clinically relevant phenotype associated with a point mutation in G_s_α and may be useful in investigations of the mechanisms of heterotopic bone formation. Together with earlier results, our findings indicate that G_s_α signalling pathways play a vital role in repressing ectopic bone formation.

## Introduction

*GNAS* encodes G_s_α, the alpha stimulatory subunit of the widely expressed heterotrimeric protein, Gs, that is required for hormone stimulated cAMP production [1]. Heterozygous loss of function mutations in *GNAS* can result in genetic disorders in which ectopic bone formation occurs [2], [3]. These include progressive osseous heteroplasia (POH), osteoma cutis (OC) and Albright Hereditary Osteodystrophy, (AHO) [4], [5], [6]. In POH ectopic bone formation in the dermis and subcutaneous fat progresses to deep connective tissue and skeletal muscle resulting in joint deformities, with mounting evidence indicating that this condition occurs predominantly on paternal inheritance of inactivating *GNAS* mutations [4], [7]. In both POH and AHO the majority of ossification is intramembranous rather than endochondral [8] but the ossification that occurs with AHO and OC tends to be superficial. Also additional features are not usually found with POH and OC. However, AHO is also characterised by short stature, round face, brachymetacarpia, dental abnormalities and neurobehavioural problems [9]. These features, and also the subcutaneous ossification, are attributable to haploinsufficiency of G_s_α in a number of tissues, and are found both on maternal and paternal transmission of the mutant allele. Thus, some patients develop AHO and others POH on paternal inheritance of inactivating *GNAS* mutations although the reasons for this are not clear.

Maternal inheritance of an inactivating *GNAS* mutation results in AHO with additional features of obesity and resistance to a variety of hormones, including parathyroid hormone (PTH) [10], [11]. These additional phenotypes can be explained in terms of the tissue specific imprinted expression of G_s_α, which is predominantly expressed from the maternal allele in specific hormone target tissues. AHO combined with hormone resistance is called PHP1a, pseudohypoparathyroidism type 1a, whereas AHO that occurs on paternal inheritance of an inactivating *GNAS* mutation without hormone resistance or severe obesity is called PPHP, pseudopseudohypoparathyroidism [11]. It is noteworthy that some patients develop POH, rather than PPHP, on paternal inheritance of an inactivating mutation in *GNAS*. Although AHO is generally associated with superficial ossification, subsets of patients have been identified with progressive ossification and PHP1a [4], [7] or PPHP. Multiple inactivating mutations have been described in humans that are associated with AHO [12], [13], [14] and POH [7], [15], [16]; all result in the reduction of the G_s_α protein, resulting in loss of GDP and GTP binding. There appears to be no genotype/phenotype correlation, and the same mutation can present with different severity and distribution of lesions both within an affected family and between different families [4]. *GNAS* is a critical negative regulator of osteogenic commitment in non osseous connective tissue [17]. It is thought that inactivation of the G_s_α protein results in cellular proliferation and differentiation and activation of osteoblasts. However the exact signalling pathways remain unknown.

**Figure 1:**
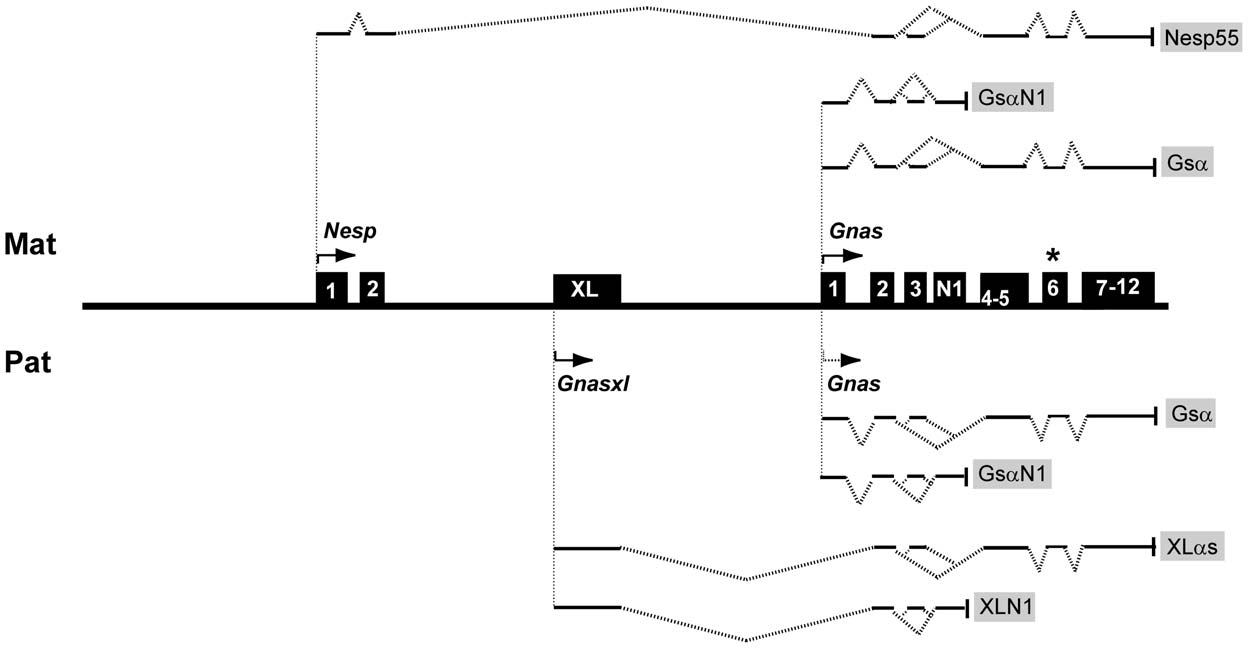
Protein coding transcript map of the mouse *Gnas* cluster (modified from ref [**66**]). Features of the maternal and paternal alleles are shown above and below the line. Arrows show direction of transcription. Transcripts arising from the first exons of protein coding transcripts, *Nesp*, *Gnasxl*, and *Gnas* splice onto exon 2 of *Gnas*. For *Gnas* the unbroken arrow shows predominant maternal expression and the dotted line indicates limited paternal expression. The asterisk indicates the *Oed-Sml* mutation in exon 6.

The orthologous mouse locus *Gnas* is also imprinted and, as in humans, G_s_α shows tissue specific imprinted expression. Although G_s_α is biallelically expressed in most tissues in the mouse it is mainly expressed from the maternal allele in renal proximal tubules, newborn brown adipose tissue, pituitary and the hypothalamus [18], [19], [20]. Furthermore the *GNAS*/*Gnas* loci are similarly organized in both species with multiple alternatively spliced protein coding transcripts arising from three different promoter regions and first exons that splice onto exon 2 of *Gnas* (Figure 1) [21], [22]. The promoter furthest downstream generates transcripts encoding G_s_α. A promoter region upstream generates transcripts for an isoform of G_s_α called XLαs which differs from G_s_α in that the first 47 amino acids are replaced by a large acidic N-terminal domain. XLαs shares many properties with G_s_α, including activation of adenylyl cyclase, thereby promoting cAMP production [23]. XLαs is paternal-specific and is only expressed in a few tissues, mainly neuroendocrine tissues [24], [25]. Neural-specific isoforms of G_s_α (GsαN1) and XLαs (XLN1) that arise from transcripts terminating in a neural exon located between exons 3 and 4 are known [26], [27]. The promoter furthest upstream generates transcripts for a chromogranin-like protein NESP55, that is exclusively maternally expressed. The open reading frame (ORF) for NESP55 is confined to its second exon, and is thus distinct from the ORF for G_s_α, and NESP55 deficient mice develop normally [28].

Mutations designed to result in loss of function of G_s_α, but leave other proteins in the cluster unaffected have been generated by targeted deletion of *Gnas* exon 1 [29], [30]. Maternal inheritance resulted in obesity [29], [30] and resistance to PTH and TSH [30] whereas paternal inheritance lead to a minimal increase in adiposity [29] and normal hormone responsiveness [30], mimicking the imprinting effects seen in PHP1A and PPHP. On both maternal and paternal inheritance shorter body length was found [30], resembling the observation of short stature in AHO, attributable to loss of biallelic expression of G_s_α.

The oedematous-small, *Oed-Sml*, mutation, derived from a specific locus mutation experiment investigating the effects of ethylnitrosourea on spermatogonial stem cells [31] is a missense mutation in exon 6 of *Gnas* resulting in a V159E substitution [32]. When maternally inherited it causes the *Oed* phenotype, characterized by extremely severe oedema at birth whereas paternal inheritance gives rise to the *Sml* phenotype of postnatal growth retardation [31]. In adult *Oed* mice the mutation also results in resistance to PTH, hypocalcemia, hyperphosphatemia [19] and obesity [33]; findings consistent with loss of function of G_s_α. Thus *Oed* constitutes a mouse model of PHP1a. On paternal transmission the *Oed-Sml* mutation will affect G_s_α, but only in tissues in which it is normally biallelically expressed. Paternal transmission will additionally affect XLαs. The neural forms G_s_αN1 and XLN1 will be unaffected. The dual effects on G_s_α and XLαs are probably reflected in the short and lean phenotype found in adult *Sml* mice [33].

We here report the finding of dermal ectopic ossification in both *Oed* and *Sml* mice in adults up to 15 months of age. This phenotype is similar on both maternal and paternal inheritance and is thus attributable to haploinsufficiency of an effect on biallelically expressed G_s_α. The ossification is confined to subcutaneous tissues and does not affect deeper tissue and so resembles the subcutaneous ossification observed with AHO. Thus the *Oed-Sml* mice provide a model of heterotopic ossification associated with loss of function mutations in *GNAS*, and may be useful in investigations of the mechanisms of heterotopic bone formation.

## Results

### Fibromatous skin masses in *Sml* and *Oed* mice

**Figure 2:**
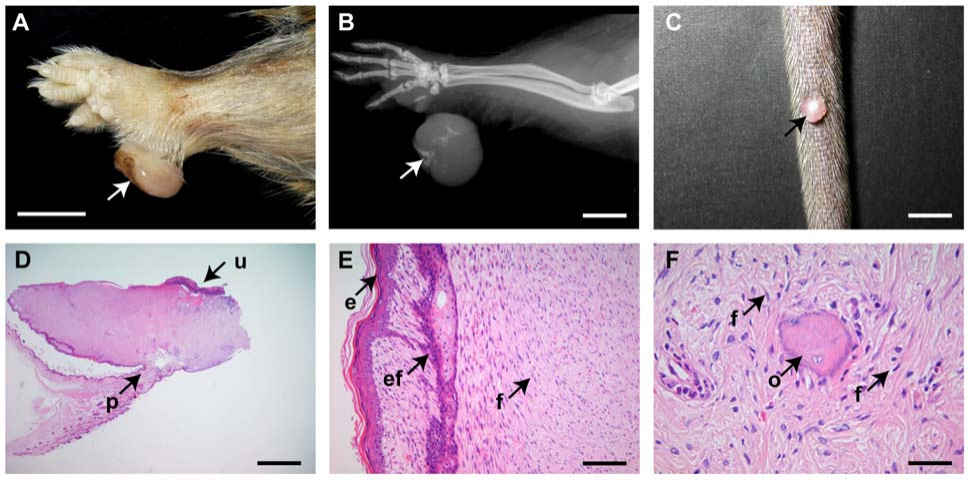
Fibromatous skin masses in *Sml* and *Oed* mice. (A) An ulcerated pedunculated hairless mass on the front paw (arrow) of 7-month-old *Oed* male mouse. (B) X-ray of this lesion shows radio-opacities (arrow). (C) A hairless nodule on the tail (arrow) of a 12-month-old *Oed* male mouse. (D, E and F) A pedunculated mass in a 8-month-old *Oed* male mouse. (D) This mass has a narrow pedicel (arrow p) and is focally ulcerated (arrow u). (E) Intact hyperkeratotic epithelium (arrow e) bordering a mass of fibromatous connective tissue (arrow f) with an entrapped follicle (arrow ef) and associated sebaceous gland. (F) Focal areas of osseous heteroplasia (arrow o) within the fibromatous mass (arrow f). Scale bars: A,B,C = 0.5 mm; D = 1 mm; E = 100 µm; F = 50 µm.

The first observed skin abnormalities in *Sml* and *Oed* mice were pedunculated or sessile hairless skin masses commonly located on the feet or tail (Figure 2A, 2C); there was only a single instance of a mass occurring elsewhere and this was on the perineum. Microscopically these skin masses were covered by sparsely haired epidermis that could be focally ulcerated (Figure 2D, 2E). Underlying tissue consisted of a fibromatous proliferation of uniform spindle cells and entrapped follicles and adnexae (Figure 2E) reminiscent of a benign fibroma of human skin [34]. Skin masses with intralesional radiographic opacities (Figure 2B) contained foci of bone that we interpret as osseous heteroplasia (Figure 2F).

These skin masses were present from 6.5 months of age onwards. During life, in *Sml* mice <10 months of age the incidence was 17% (6/35) but no skin masses were seen in 15 +/+ sibling controls. In *Sml* mice >12 months of age the incidence rose to 74% (39/53), significantly different from both the complete lack of skin masses in 19 male +/+ sibling controls >12 months, (*P*<0.0001 Fisher's exact test) and in *Sml* mice <10 months of age (*P*<0.0001Fisher's exact test). In *Oed* mice <10 months of age the incidence was 25% (5/20) significantly different (*P*<0.05) from the absence of skin masses in 20 +/+ sibling controls. In *Oed* mice >12 months of age the incidence was 52% (11/21), significantly different from the complete absence of skin masses in 13 +/+ sibling controls (*P*<0.01) but not significantly different from the incidence at <10 months. The incidences of skin masses were not significantly different in *Sml* and *Oed* at <10 months or >12 months of age.

### Widespread radiographic lesions in the skin

**Figure 3:**
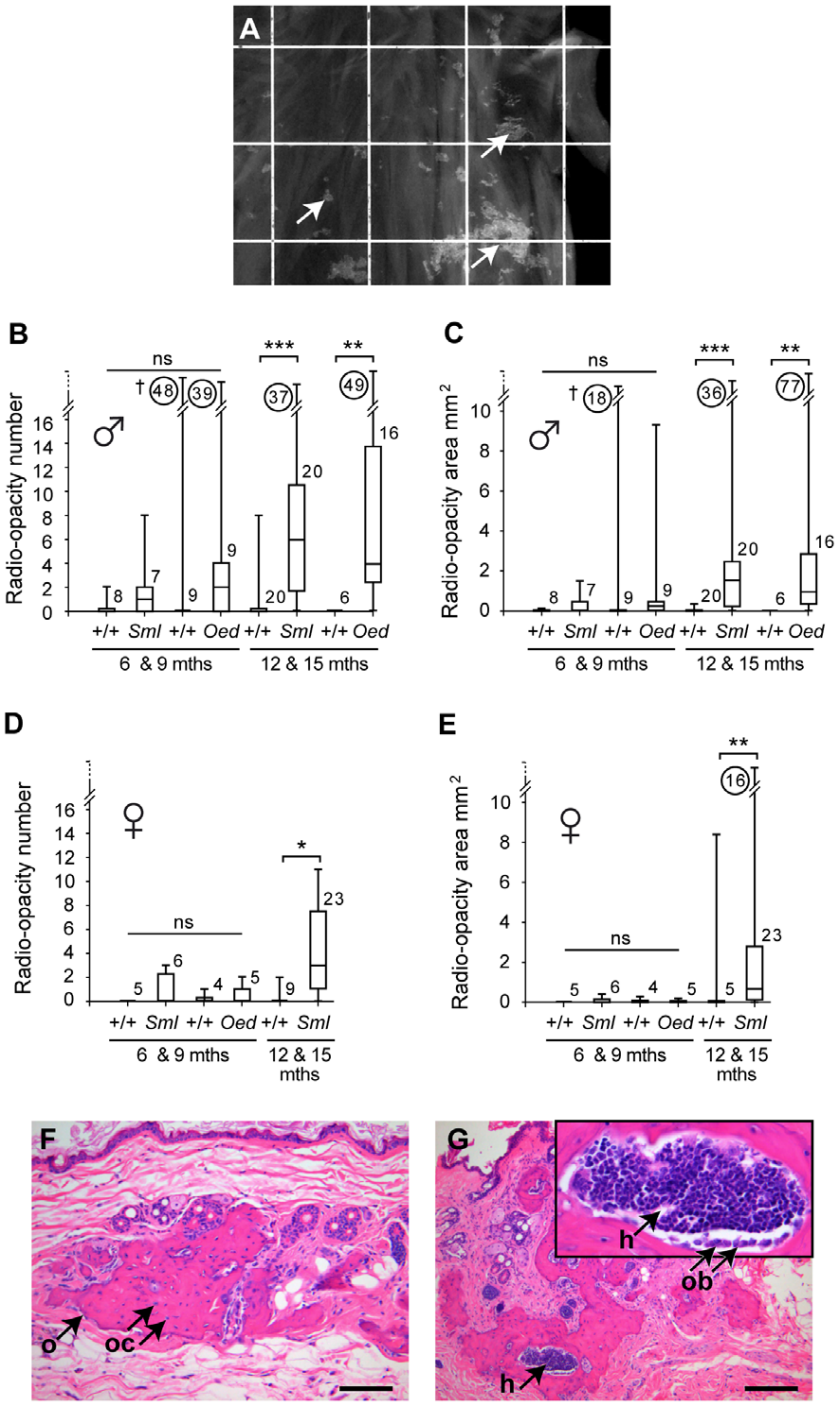
Skin radio-opacities increase with age in *Sml* and *Oed* mice and correspond to foci of osseous heteroplasia. (A) X-ray image of radio-opacities in the skin of a 12-month-old *Sml* male imaged on a 1×1 cm grid. (B) The number and (C) cumulative area of skin radio-opacities in *Sml* and *Oed* male mice at 6 & 9 months of age are not significantly different from +/+ controls, however they are significantly increased in the 12 & 15-month-old cohorts compared to +/+ mice. (D) The number and (E) cumulative area of skin radio-opacities in *Sml* and *Oed* females at 6 & 9 months of age are not significantly different from +/+ controls, however they are significantly increased in the 12 & 15-month-old *Sml* compared to +/+ controls. In the box and whisker plots each box represents the median with 25 and 75% inter-quartile ranges, with whiskers representing the data range (minimum and maximum). The group size *n* is indicated alongside each histogram bar, whereas the circled numbers are the off-scale maximum values. ns = not significant *P*>0.05, * *P*<0.05, **P<0.01, ***P<0.001. Kruskall Wallis non-parametric one-way ANOVA tests were performed with Dunn's multiple comparison tests for post hoc testing. † A single male +/+ control with high levels of radio-opacities was an outlier that had a wounded skin. (F). Radio-opacities correspond to foci of osseous heteroplasia (arrow o) with osteoclasts (arrow oc) beneath intact skin. (G) Focus of hematopoietic tissue (arrow h) within an area of osseous heteroplasia, inset is enlarged view (arrow h), the bone surface has an osteoblast layer (arrow ob). Scale bars: F = 100 µm; G = 200 µm.

To explore whether osseous heteroplasia was confined to the skin masses we took whole body x-rays and found radio-opacities were more generalized. To determine whether radio-opacities were superficial (in the dermis or subcutaneous fat) or deep (in connective tissue and muscle) whole body radiographs were obtained before skin dissection in a subset of 40 *Sml* and *Oed* mice aged 12 and 15-months (18 *Oed* males, 15 *Sml* males and 7 *Sml* females). In all cases there were no detectable adhesions between the skin and underlying trunk and limb muscles and the skin dissected cleanly. Furthermore the radio-opacities were accounted for in the dissected skin (Figure 3A) and not in trunk or limb musculature. In 6 and 9 month *Sml* and *Oed* male mice, the radio-opacity number (range 0–48) and cumulative area (range 0–18 mm^2^) overlapped and did not significantly differ (*P*>0.05) from their respective +/+ controls (Figure 3B and 3C). A single outlier +/+ male with wounded skin gave the high value of 48 lesions with a cumulative area of 18 mm^2^. At 12 and 15 months radiographic changes had risen significantly (*P*<0.001) in *Sml* males (range 0–37 and 0–36 mm^2^) over +/+ controls (range 0–8 and 0–0.3 mm^2^). In *Oed* males radiographic changes (range 0–49 and 0–77 mm^2^) were significantly higher (*P*<0.01) than +/+ controls (zero for both parameters) (Figure 3B and 3C).

In *Sml* and *Oed* female mice, the number (range 0–3) and cumulative area (range 0–0.4 mm^2^) of radio-opacities were low and not significantly different (*P*>0.05) from their respective +/+ controls at 6 and 9 months. However, by 12 and 15 months, both radiographic changes had risen in *Sml* females (range 0–11 and 0–16 mm^2^) significantly (*P*<0.05, and *P*<0.01 respectively) above +/+ control levels (range 0–2 and 0–8 mm^2^) (Figure 3D and 3E).

### Radiographic lesions in the skin correspond to foci of osseous heteroplasia

Skin histology revealed that areas with radio-opacities (Figure 3A) corresponded to foci of subcutaneous and dermal osseous heteroplasia characterized by irregular bone lamellae with osteoclasts within lacunae (Figure 3F). Some of the bone foci had large lacunae lined with osteoblasts and accumulations of hematopoietic tissue (Figure 3G). When skin samples from *Sml* and *Oed* mice with radio-opacities were systematically examined for underlying histological changes, 60% (6/10) and 91% (32/35) were confirmed to have foci of dermal or subcutaneous ossification in 6 & 9 month-old and 12 & 15 month-old cohorts respectively (Table 1). In contrast, mineralization of dermal or subcutaneous connective tissue characterized by basophilic extracellular collagenous stroma was a less common finding than bone in *Sml* and *Oed* 12 & 15 month-old mice (Table 1). Dermal or subcutaneous bone was found in significantly fewer 12 & 15 month *+/+* mice compared with *Sml* and *Oed* mice (11% [3/27] versus 92% [35/38]; *P*<0.0001) (Table 1). In some *+/+* mice, bone was associated with injury to overlying skin whereas the majority of instances of dermal bone in *Sml* and *Oed* mice occurred beneath intact uninjured epithelium (Figure 3F, 3G). Sixteen *Sml* and *Oed* mice from 6 & 9 and 12 & 15 month old cohorts that had confirmed dermal or subcutaneous bone underwent a systematic histological examination of internal organs and revealed unremarkable low background incidence of bony change and mineralization. One mouse had small foci of bone in the lung, 4 had minimal focal mineralization in various organs including the kidney, Harderian gland, stomach, intestines and male accessory reproductive gland. The finding of late onset osseous heteroplasia on both paternal and maternal inheritance of the *Oed-Sml* mutation suggests that these lesions are due to haploinsufficiency of G_s_α.

**Table 1:** Histological analysis of skin x-ray radio-opacities in *Oed* and *Sml* mice.

		skin x-ray	histology	
			osseous	
		radio-opacity	heteroplasia	mineralization
6 & 9 month	+/+ (*n* = 22)	3	0	0
	*Sml* (*n* = 10)	5*	2^ns^	1
	*Oed* (*n* = 13)	5^ns^	4*	1
12 & 15 month	+/+ (*n* = 27)	3	3	1
	*Sml* (*n* = 19)	16***	16***	1^ns^
	*Oed* (*n* = 19)	19***	16***	2^ns^

2×2 Fisher Exact tests were used to compare proportions of *Sml* or *Oed* with +/+ control mice bearing each lesion. ns = not significant *P*>0.05,

* *P*<0.05,

*** *P*<0.001.

### Plasma biochemistry

In young adult *Oed* mice the *Gnas* mutation results in resistance to PTH, hypocalcemia and hyperphosphatemia [19]. We therefore examined whether similar changes occurred in older *Oed* mice and in *Sml* mice, since they could conceivably play a role in osseous heteroplasia.

**Figure 4:**
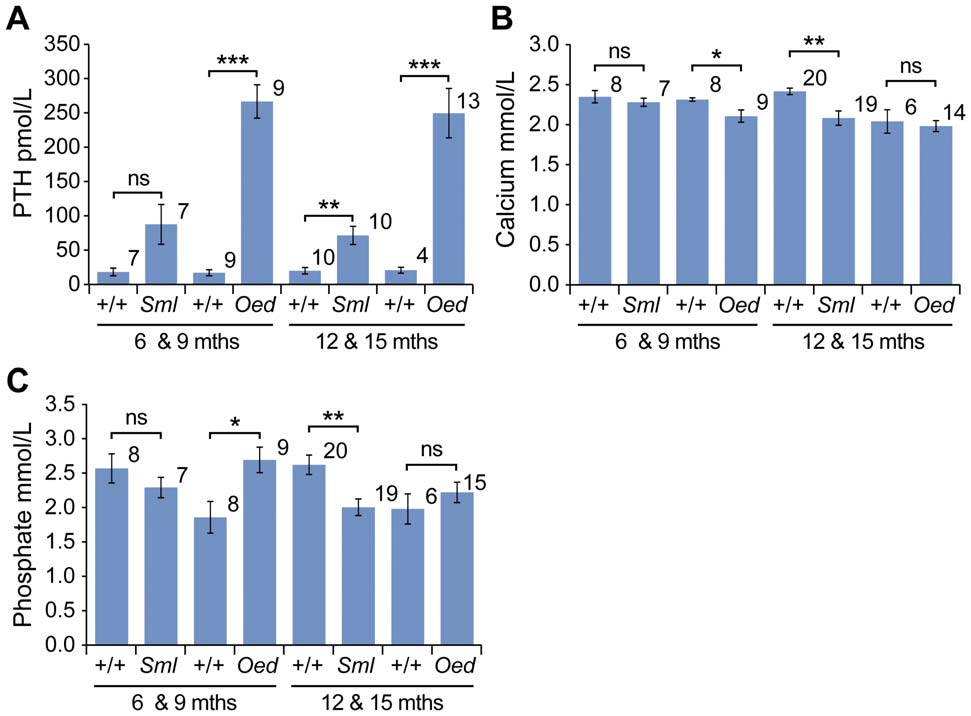
Plasma biochemistry phenotypes of male *Sml* and *Oed* mice. (A) Plasma PTH was significantly elevated in *Oed* mice at 6 & 9 and 12 & 15 months of age compared to +/+ controls. The modest elevation in *Sml* PTH levels at 6 & 9 months failed to reach significance (*P* = 0.053) but PTH was significantly elevated at 12 & 15 months compared with respective +/+ controls. Plasma calcium corrected for albumin levels (B) in *Oed* mice was lower than their respective +/+ controls at 6 & 9 months but not at 12 & 15 months. Calcium in *Sml* males was not lower than in their respective +/+ controls at 6 & 9 months but was lower than controls at 12 & 15 months. Plasma phosphate levels (C) in *Oed* mice were higher compared with their respective +/+ controls at 6 & 9 months but not at 12 & 15 months. Plasma phosphate in *Sml* males was not significantly different from +/+ controls at 6 & 9 months and at 12 & 15 months was lower than +/+ controls. The group size *n* is indicated alongside each histogram bar, the error bar is ± s.e.m; ns = not significant *P*>0.05, *P<0.05, **P<0.01, ***P<0.001. 2-tailed t-tests with unequal group variance were performed on the PTH data.

In *Oed* males PTH was highly elevated at 6 & 9 months (266±24 pmol/L versus 17±4 pmol/L; *P*<0.0001) and at 12 & 15 months (249±36 pmol/L versus 22±5 pmol/L; *P*<0.0001) compared with +/+ controls. The moderate elevation in PTH in *Sml* males did not achieve statistical significance at 6 & 9 months (88±29 pmol/L versus 18±5 pmol/L; *P* = 0.054) but at 12 & 15 months was significant (71±13 pmol/L versus 19±5 pmol/L; *P*<0.002) (Figure 4A).

Plasma calcium corrected for albumin in *Oed* males was significantly lower than their respective +/+ controls at 6 & 9 months (2.1±0.08 mmol/L versus 2.3±0.02 mmol/L, *P* = 0.029), but not at 12 & 15 months. Calcium in *Sml* males was not significantly lower than in their respective +/+ controls at 6 & 9 months but was lower than controls at 12 & 15 months (2.1±0.09 mmol/L versus 2.4±0.04 mmol/L, *P* = 0.0021).

Plasma phosphate levels in *Oed* males were significantly higher compared with their respective +/+ controls at 6 & 9 months (2.7±0.18 mmol/L versus 1.9±0.23 mmol/L, *P* = 0.014), but not at 12 & 15 months. Plasma phosphate in *Sml* males was not significantly different from +/+ controls at 6 & 9 months and at 12 & 15 months was actually lower than +/+ controls (2.0±0.12 mmol/L versus 2.6±0.14 mmol/L, *P* = 0.0021).

## Discussion

In this animal model we have identified both radiological and pathological evidence of heterotopic ossification or osteoma cutis (the formation of mature bone in soft tissues). In this study we have analysed two cohorts of 6 & 9 month (*n* = 52) and 12 & 15 month (*n* = 102) *Oed/Sml* mice and their +/+ controls. In summary we identified: (a) a statistically significant increase in soft tissue lesions (histologically confirmed as fibroepithelial polyps) in aged *Sml* and *Oed* mice; (b) a statistically significant increase in both the number and cumulative area of radio-opacities in aged *Oed* and *Sml* male and female mice; and (c) highly elevated PTH levels in both younger and older *Oed* mice and moderately elevated PTH levels in older *Sml* mice.

The first observed skin phenotype, benign cutaneous fibroepithelial polyps, is similar on both maternal and paternal inheritance of the *Oed-Sml* mutation and are thus attributable to haploinsufficiency of biallelically expressed Gsα. These fibromas have some resemblance to the skin angiofibromas described by Sakamoto et al. [35] in their exon 2 *Gnas* deletion model.

It is interesting to note that the fibromas described in the latter model were stated to arise in a similar distribution to ours on the ear, paws and tail, occurred with a similar age and sex distribution, and were associated with calcification. Sakamoto et al. [35] state that pre-existing trauma may be a predisposing factor, which may be a similar cause in our model considering the similarities in lesional distribution, the only difference being we did not find fibromas on the ears in our model despite inadvertent injury to the ear caused by notching for identification/genotyping.

In the Sakamoto et al. paper [35] all lesions were associated with calcification only, while in our study, we identified three lesions that were associated with ossification. However we did not serially section all lesions to confirm the presence or absence of microscopic ossification in others. It is unclear whether these lesions represent a subset of dermal ossification lesions that became pedunculated or were associated with additional fibrous proliferation, or whether they represent a separate entity with additional etiology.

Although we identified a statistically significant increase in soft tissue polypoid lesions in this model, the clinical significance is unclear. In our review of the clinical literature of AHO and PHP1A patients there is no mention of soft tissue lesions as described by us or by Sakamoto et al. [35].

The second phenotype, namely subcutaneous ossification and calcification, was observed both radiologically and confirmed by histological examination. Previous mouse models of inactivating *Gnas* mutations generated via a variety of mechanisms have also shown evidence of superficial heterotopic ossification [17], [36], [37] with similar histological features to those found in our model. In a conditional renin induced exon 1 *Gnas* deletion model, skin mineralisation plus additional skeletal deformities were observed in both male and female mice with local deletion of *Gnas* in both the kidney and extrarenal tissues including skin [36]. In a separate targeted exon 1 deletion model generated by Huso et al. [37], heterozygous male and female mice developed non-progressive subcutaneous ossifications related to sites of trauma with ageing. In our study, we also identified late onset heterotopic ossification confined to subcutaneous tissues on both maternal and paternal inheritance of the *Oed-Sml* mutation. Overall, heterotopic ossification is similar in both *Oed* and *Sml* indicating that the difference in genetic background has not had a significant effect on the phenotype. The *Oed-Sml* mutation will affect biallelically expressed G_s_α in both *Oed* and *Sml*, imprinted G_s_α in *Oed*, and imprinted XLαs in *Sml*. The similarity in the ossification phenotype in both *Oed* and *Sml* indicates that it is attributable to partial loss of G_s_α in tissues in which *Gnas* is biallelically expressed, not to imprinted G_s_α or XLαs. Thus the ENU mutation resulting in *Oed* and *Sml* gives rise to a bone phenotype more in keeping with that generally associated with AHO (both PHP1A and PHPP) rather than POH, in that it is confined to superficial tissues and is attributable to haploinsufficiency of biallelically expressed G_s_α.

Patients with PHP1A develop PTH resistance and associated hypocalcemia and hyperphosphatemia, features attributable to imprinted expression of *GNAS* [3]. *Oed* mice with a maternally derived mutant *Oed-Sml* allele were known to have highly elevated PTH levels at 10 weeks of age [19], and have now been shown to have highly elevated PTH levels up to 15 months of age. *Gnas* shows imprinted expression in proximal renal tubules, the primary site of PTH action in the kidney [18], [19], [30], [38], [39]. However, in imprinted tissues *Gnas* appears to be predominantly but not exclusively expressed from the maternally derived allele with about 70–80% expression from the maternal allele and 20–30% from the paternal allele [18], [38], [40]. It had also been shown previously that *Oed* mice that additionally carried a mutation leading to loss of imprinting of *Gnas* so resulting in biallelic expression of *Gnas* did not have, as expected, a wild type concentration of PTH but one that was intermediate between wild type and *Oed* [19]. To account for this intermediate level it was proposed that whereas the now functional paternal allele enabled normal PTH signaling the mutant maternal allele did not. Thus the *Oed-Sml* allele probably generates a mutant Gsα polypeptide that can associate with Gsβ and Gsγ polypeptides to form a heterotrimeric Gs protein but one that that is non functional, and so PTH signaling activity is restricted resulting in a partial normal response to PTH [19].

A similar argument for a partial normal response to PTH can be put forward to account for the moderate elevation in PTH concentration in *Sml* mice in the present study. In these mice it is expected that 70–80% of the *Gnas* expression in proximal renal tubules will be from the wild type functional maternal allele and 20–30% from the mutant nonfunctional paternal allele. The wild type maternal allele will enable normal PTH signaling but the mutant paternal allele will not, so curtailing overall signaling activity through Gsα and accounting for some elevation in the PTH level but to a much lesser extent than in *Oed* mice.

Although hypocalcemia and hyperphosphatemia were found to be associated with the highly elevated PTH levels at 10 weeks of age in *Oed* mice [19] and now at 6 & 9 months in this study, hypocalcemia and hyperphosphatemia did not occur at 12 & 15 months in *Oed* mice and the reason(s) for this are unclear. Although hypocalcemia was associated with the moderate elevation in PTH in *Sml* mice at 12 and 15 months, hyperphosphatemia was not seen and indeed the mice were hypophosphatemic relative to their respective controls, and the reason(s) for this is are not clear.

It should be noted that the average plasma calcium (2.1–2.4 mmol/L) and phosphate (1.9–2.7 mmol/L) levels for all cohorts in this study are close to MRC Harwell baselines found in 18 week old mice in other inbred mouse strains (C57BL/6, BALB/c, C3H and 129) (see data deposited in http://europhenome.org). Although *Oed* and *Sml* mice are on a different genetic background to these inbred strains and are older, the observation that plasma calcium and phosphate levels found in *Oed* and *Sml* mice are within normal limits for other strains of mice raises the question whether the relative degree of hypocalcaemia and hyperphosphatemia is either pathological or contributory to the development of osseous heteroplasia. The absence of any significant degree of dermal mineralization in *Oed* and *Sml* mice suggests that the changes in are not in themselves pathological. Taken together, tissue calcification does not occur as a direct consequence of PTH resistance nor does tissue calcification appear to be critical to the development of osseous heteroplasia in *Oed*, and *Sml* mice.

We have reviewed the clinical literature of patients with AHO or PHP1a who have had additional mutational analysis (Table S1). Of a total 120 patients with either syndrome reported in the literature, 61 were stated to have superficial subcutaneous calcifications which were either multiple or single plaque like lesions, located frequently on the abdomen, back, limbs and digits (Table S1). Some calcifications were reported to be associated with ossification; in other patients osteomas were identified as well as subcutaneous calcification. Rarely intracerebral calcification was reported but there was no reference to either progressive or deep calcification of other organs in these patients. Of significance, our review of 120 patients reported in the literature with either AHO or PHP1a and mutational analysis revealed that there was no report of subcutaneous fibromas or fibroepithelial polyps in any of the patients investigated. In one patient there is reference to a calcifying aponeurotic fibroma with central osteoid formation which may have in fact been a polypoid osteoma [41]. It appears therefore that there is no correlation between fibroma observed in our model and that of Sakamoto et al. [35] and the human clinical syndromes AHO and PHP1a [4], [7], [12], [41]–[63] (Table S1).

A wide range of genetic mutations were identified in these patients ranging from insertion and deletions of base pairs leading to frameshift mutations, premature termination and truncated proteins to missense mutations in which a particular amino acid was substituted. There was no clear genotype-phenotype correlation, though there appeared to be two mutation hotspots in exon 7 and exon 10. Although there are no reported patients with the exact missense mutation created in the *Oed/Sml* mouse (V159E), there are reports of two patients with other missense mutations in exon 6 (Table S1). One mutation is in codon 159, V159M in a patient with PHP1a but no reported subcutaneous calcification [63] and the other mutation is a substitution of cysteine for arginine (R165C) in codon 165 in a patient with PHP1a with reported subcutaneous calcification [12] (Table S1).

In summary our data confirm some of the cutaneous findings associated with other targeted mouse models of AHO but is exceptional in that it has both osseous heteroplasia and fibromas and is caused by a missense mutation. Our model is unique in that it is the first model described with a clinically relevant phenotype associated with a point mutation and thus will prove invaluable in dissection of the genotype-phenotype correlation in the complex array of human genetic mutations that result in AHO/PHP1A. Together with earlier results, our findings indicate that G_s_α signalling pathways play a vital role in repressing ectopic bone formation. Thus the *Oed-Sml* mice provide a model of heterotopic ossification associated with loss of function mutations in *GNAS*, and may be useful in investigations of the mechanisms of heterotopic bone formation.

## Materials and Methods

### Ethics statement

Full details of the study were approved after review by MRC Harwell ethical review committee and the humane care and use of mice in this study was carried out under the authority of the appropriate UK Home Office Project Licence.

### Mice

The generation and husbandry of *Gnas^Oedsml-pat^*, *Gnas^Oedsml-mat^* and their wild-type litter mates *Gnas^+/+^* (hereafter referred to as *Sml*, *Oed* and *+/+* respectively) is described by Kelly et al. [33]. Briefly, paternal inheritance of the mutation results in *Sml* mice, and maternal inheritance results in *Oed* mice and both show relatively poor viability [31]. *Sml* can be maintained on our standard laboratory stock, 3H1A and *Oed* on *M.m.castaneus* (MCA). *Sml* and *Oed* cannot be maintained on the same background because *Oed* is lethal on 3H1A and *Sml* is lethal on MCA. Thus *Sml* mice were generated by crossing 3H1A females with *Sml* males and *Oed* mice by crossing *Sml* females with MCA males.

Initially *Sml* and *Oed* males aged to 6, 9, 12 and 15 months were used but subsequently females were incorporated into the study. Data for 6 & 9 month-old cohorts and 12 & 15 month old cohorts were each pooled to enlarge group size and aid statistical analyses.

Specific pathogen-free mice were housed in individually ventilated cages under a 12-h light/12-h dark cycle, temperatures of 21±2°C and humidity of 55±10%. Mice were fed an expanded rat and mouse no. 3 breeding diet (Special Diets Services, Witham, UK) containing 1.15% calcium, 0.82% phosphate and 4089 units/kg vitamin D, and given water ad libitum. Cohorts of mice were aged to 6, 9, 12 and 15 months for pathology analysis and plasma biochemistry. Skin lesions that were visible during life were recorded.

### Histology and image analysis

Macroscopic skin masses from the paws and tails and samples of skin corresponding to identified radio-opacities were processed for histology. In mice with no detectable radio-opacities, three randomly chosen interscapular skin sections corresponding to the site surveyed in skin X-ray analysis were used to assess the possibility of microscopic dermal changes. The histology of all major internal organs (brain, salivary glands, ears, eyes, Harderian gland, reproductive tract, heart, lungs, trachea, esophagus, stomach, liver, intestine, pancreas, kidneys, adrenals, thyroids, thymus, spleen and mesenteric lymph node) were examined in a subset of *Sml* and *Oed* mice. Tissues were fixed in 10% neutral buffered formalin, processed and embedded in paraffin wax and 3 µm sections were stained with Haematoxylin and Eosin using standard techniques [64]. Digital images were captured on an Olympus BX51 microscope using ×20, ×40 or ×60 Plan Achromat objectives with neutral density filter, on a ColorView Soft Imaging System software using automatic exposure times.

### X-ray analysis

Euthanized mice were fixed flat in 10% buffered formalin after removal of thoracic and abdominal viscera. The skin was dissected from underlying subcutaneous fat and muscle in one piece to include any gross skin lesions of the paws and tail. The skin was thoroughly washed in tap water to remove any adherent material then placed on a 1 cm^2^ grid to obtain life-size dorsoventral digital X-ray images with a MX-20 Faxitron using a 26 Kv and a 3 second exposure. The entire skin was initially surveyed for radio-opacities and soft tissue masses in a series of images covering head and neck, trunk, pelvis and tail, and limb distal extremities. A 3×3 1 cm^2^ grid area covered the dorsal mid shoulder to lumbar region, and a ‘X’ shaped pattern of five of these 1 cm^2^ grid squares were chosen to count the number and cumulative area of radio-opacities (mm^2^). The total number and area of radio-opacities in each skin was measured using Photoshop CS3 extended edition (Adobe systems, USA).

### Blood collection

Blood was collected from the jugular vein of mice under terminal anesthesia induced by an i.p. overdose of sodium pentobarbital. Lithium-heparin plasma samples were aliquoted and frozen at −80°C.

### Clinical biochemistry and PTH assay

Plasma was assayed for calcium, phosphate and albumin on an Olympus AU-400 analyzer. Parathyroid hormone was measured using a mouse intact PTH ELISA kit (Immutopics). Calcium was corrected for albumin levels using the standard formula [65]: corrected calcium = measured calcium−[(albumin g/l−30)×0.017].

### Statistics

D'Agostino & Pearson omnibus normality tests were performed on radio-opacity and biochemistry data sets. The data was not normally distributed for the radio-opacity data therefore Kruskall Wallis non-parametric one-way ANOVA tests were performed followed by Dunn's multiple comparison tests for post hoc testing. In the biochemistry data t-tests were performed comparing *Sml* and +/+ controls, and *Oed* and +/+ controls at each age. Fisher exact tests were used for the presence or absence of skin radio-opacities and macroscopic skin lesions. Data is presented as mean ± standard error of the mean (s.e.m.), or using box and whisker plot; each box represents the median with 25 and 75% inter-quartile ranges, with whiskers representing the data range (minimum and maximum).

## Supporting Information

Table S1 **Paper review of SCO and genetic mutations in AHO/PHP1a.**
(XLSX)
